# Global Research Trends and Hotspots on Submarine Groundwater Discharge (SGD): A Bibliometric Analysis

**DOI:** 10.3390/ijerph17030830

**Published:** 2020-01-29

**Authors:** Qian Ma, Yan Zhang

**Affiliations:** 1 Tianjin University Library, Tianjin University, Tianjin 300350, China; 2 Information Science Institute, Tianjin University, Tianjin 300350, China; 3 State Key Laboratory of Biogeology and Environmental Geology and School of Water Resources and Environment, China University of Geosciences-Beijing, Beijing, 100083, China; yanzhang@cugb.edu.cn

**Keywords:** submarine groundwater discharge (SGD), bibliometric, marine environment, hydrological cycle

## Abstract

Submarine groundwater discharge (SGD), a major component of the hydrological cycle, has significant impacts on the sustainable development of the marine environment. This study aimed to examine the literature characteristics and research hotspots of SGD based on Web of Science’s citation database from 1998–2019. With systematic bibliometric analysis, insights were made into multiple aspects including research output, subject categories, journals, countries/territories, institutions, authors, and hotspots and research trends. Results showed that the current amount of publications on SGD has increased exponentially. The characteristics of multi-subject, active international and inter-institutional collaborations were identified. There were 11 core journals publishing the research on SGD, and the number of covered journals increased linearly from 1998. USA had distinct advantages in publication outputs and took the core position in international collaborations. At present, the research hotspots of SGD mainly include the following: dynamics process and estimation of SGD with hydrogeological methods, tracer techniques, geochemical process in subterranean estuary, and dissolved material inputs to coastal waters via SGD. Citation analysis implied much development space in carbon flux transported by SGD and the implement of head as groundwater tracer. These results provided an instructive perspective of the present situation and future research direction on SGD.

## 1. Introduction

Submarine groundwater discharge (SGD) refers to any and all flow of water on continental margins from the seabed to the coastal ocean, i.e., the groundwater exchange between land and the sea in short [1]. SGD has been recognized as an important pathway for nutrient, contaminants, and other chemical materials transport to coastal water, which has significant impacts on the sustainable development of marine environment and geochemical cycles [2,3,4]. 

As an important but hidden input pathway, SGD was neglected for many years for the great difficulty in quantification. Few researches were made before the 1990s, such as Johannes and Hearn [5] observed the nutrient and salinity regimes in a coastal lagoon under the influence of SGD. Since the study of SGD quantification published in Nature [6], processes associated with SGD have attracted more and more attention, and have gradually become a research hotspot in coastal areas. It was widely reported that SGD provides a large amount of important dissolved mass input to marine environment, including carbon, nutrient and microelements that marine plants and animals need [7,8]. The order of magnitude of SGD was estimated to be 10 to 10^3^·m^3^d^−1^m^−1^, which is considerable with the long coastlines worldwide [5,9,10,11,12]. Owing to the growing groundwater pollution in recent decades, SGD often contains high concentrations of various contaminants such as nutrients, organic compounds, radionuclides and heavy metals. The impacts of SGD on marine environment and ecology are becoming increasingly significant [3,13,14,15]. Especially, the nutrient inputs via SGD could rival those via river water in some areas, which is critical to the nutrient recycling in marine ecosystems [16,17,18,19]. Besides that, marine disasters, such as eutrophication and algae bloom, were always found to be closely related to nutrient inputs via SGD [20]. 

Bibliometrics is a useful tool for quantitatively evaluating the present situation and development of scientific production in specific study fields [21,22]. It has been applied in various fields such as solid waste [23], compute science [24], clinical medicine [25], proteomics [26], and river water quality [27]. The traditionally analyzed indicators included publication outputs, subject categories, authors, journals, contributing countries and institutions. *h*-index, first proposed by Hirsch [28], is a representative indicator of productivity and impacts in evaluating scientific capability of author, journal, country and institution. The evaluation of countries and institutions performance developed into five indicators recently, including total, first-author, corresponding-author, and collaborative publications [29,30,31]. The analysis of keywords has been used to figure out hot topics and research trends in recent years [32,33].

This paper aimed to provide a systematic analysis of research on SGD during 1998–2019 via bibliometric technique. The goal of this paper included: (1) identifying characteristics of research outputs, subject categories, journals and core authors in SGD research; (2) evaluating the research performance of country and institution from multi-perspectives; (3) highlighting the hotspots and research trends, which may provide useful guidance for future research. The results of analayses are helpful in realizing the research progress and frontier dynamics of submarine groundwater discharge (SGD).

## 2. Materials and Methods 

### 2.1. Data Source

The data were retrieved from the Science Citation Index Expanded (SCIE), the Clarivate Analytics’ Web of Science. SCIE database is a comprehensive database mainly focusing on basic science including natural science, biology, and medicine and so on. It is the most commonly used data source for bibliometric studies [34]. According to 2018 Journal Citation Reports, SCIE database covers more than 12,000 significant journals of science and technology across 254 subject categories, with new records being updated every day. The retrieval strategy is mainly determined by three retrieval terms: marine, groundwater and discharge. Following topic terms were used in retrieving SGD-related publications in SCIE database from 1998 to 2019: (1) (“*marine groundwater” or “*marine ground water”) and discharg*; (2) (“*marine groundwater” or “*marine ground water”) and “SGD”; (3) (“groundwater discharg*” or “ground water dicharg*” or “groundwater exchang*” OR “ground water exchang*” or “pore water discharg* rate*” or “pore water exchang* rate*”) and (“*marine” or “sea” or “ocean” or “bay” or “coast*” or “beach*” or “gulf” or “inlet” or “subterranean estuary”). These synonyms listed above could help retrieve the vast majority of SGD-related publications. Data cleaning was performed with duplicate and irrelevant data removed through initial screening of the title, abstract and keywords. 

### 2.2. Data Analysis

A total of 1844 publications were obtained with following aspects analyzed intensively: (1) characterization of research outputs; (2) distribution of subject categories; (3) core journals; (4) geographic distribution and collaboration performance of country/territory; (5) research performance of institution; (6) author profile; (7) hot topics and research trends. In this study, systematic bibliometric techniques were employed including citation analysis, social network analysis, and co-word cluster analysis. In citation analysis, citation counts, citations per publication and *h*-index were used to evaluate academic influence. Social network analysis was applied to show national cooperation network. Co-word cluster analysis and word frequency analysis were utilized to analyze the hot topics and global research trends. HistCite, a literature index analysis software, can help to quickly master key papers in a certain field based on citation analysis. Vosviewer software was utilized in social network analysis (SNA) and co-word cluster analysis.

## 3. Results and Discussion

### 3.1. Research Output Trend

**Figure 1:**
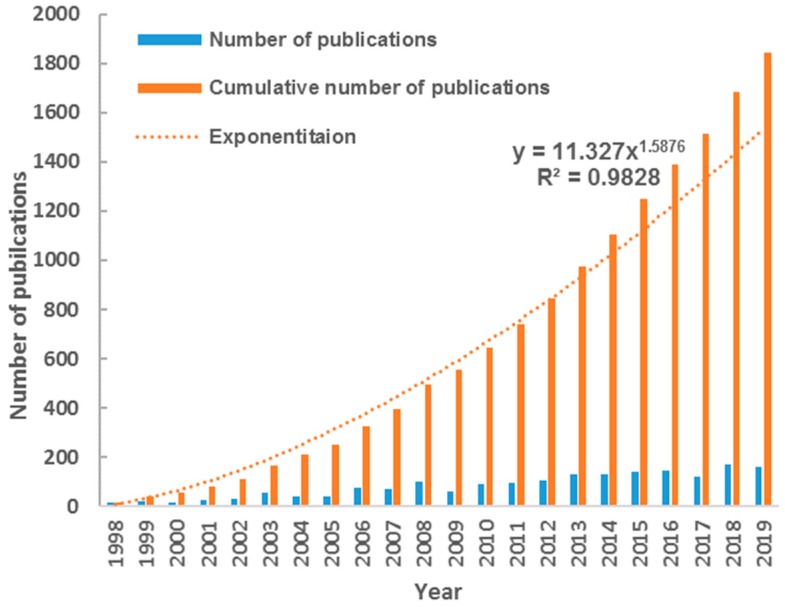
Development trend of SGD-related publications.

Figure 1 shows the development trend of SGD-related publications each year. It demonstrates that the number of publications increased significantly from 18 in 1998 to 160 in 2019 with an annual growth rate of 15.39% on average. The cumulative number of publications fits Price’s curve with R^2^ = 0.98, indicating the exponential growth of cumulative amount of SGD research. Since 2013, the actual value of the cumulated number has obviously exceeded the theoretical fitting value, suggesting the large potential for SGD research. 

### 3.2. Distribution of Subject Categories

**Figure 2:**
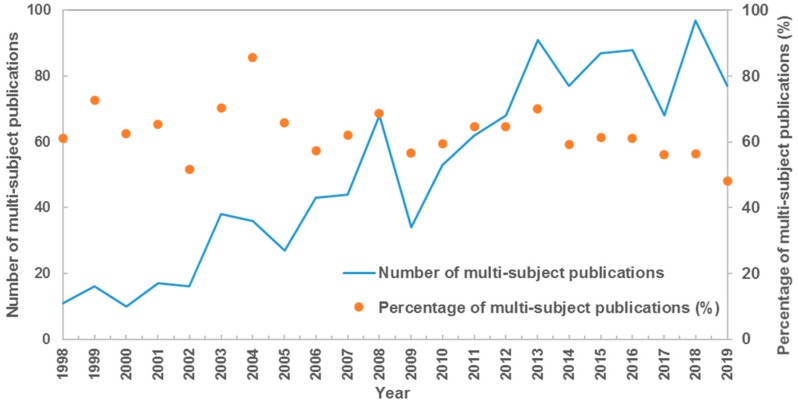
Number of multi-subject publications and its percentage.

The subject categorization scheme classified by the Web of Science database was used to analysis the distribution of subject category, with each paper assigned to at least one subject category. The 1844 SGD-related publications covered 55 Web of Science categories. The top 5 main subject categories were Environmental Sciences (563), Water Resources (484), Geosciences, Multidisciplinary (474), Oceanography (459) and Marine & Freshwater Biology (292), with almost 81% of total publications included. There were 1128 multi-subject publications (i.e., paper belonged to more than one subject category), accounting for 61% of total output. For the top 10 hot subject categories, as shown in Table 1, the percentages of multi-subject publications were all larger than 75% except Geochemistry & Geophysics. Environmental Sciences was the main collaborative subject category, which had collaborated with other 28 subject categories of 439 multi-subject publications in total. Furthermore, the number of multi-subject publications increased stably in total, with its percentage share always larger than 50% in each year (Figure 2). These results reflected the character of active multi-discipline cross in SGD research. 

### 3.3. Journals

**Figure 3:**
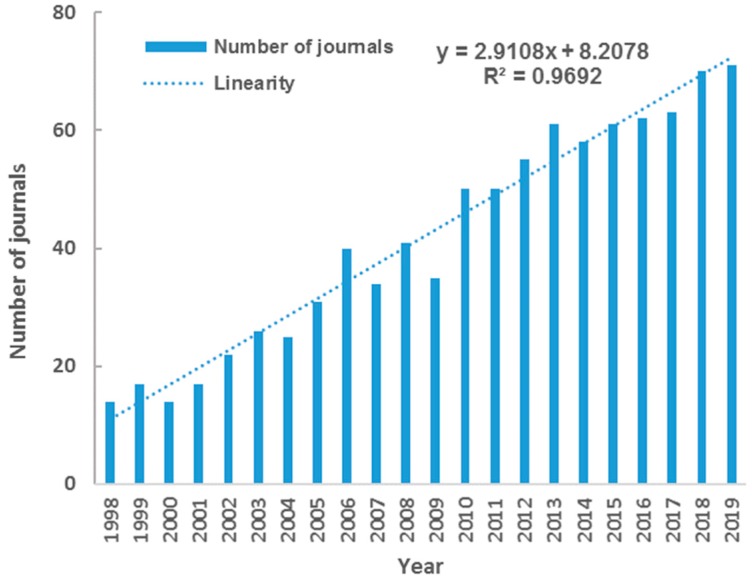
The growth dynamics of journals covered by SGD research.

Overall, 310 academic journals have published publications related to SGD. As illustrated in Figure 3, the journals covered by SGD research expanded stably. The number of journals published SGD research showed an almost linear growth, from 14 in 1998 to 71 in 2019. 

According to Bradford’s law, journals in a research topic can be divided into three parts: the core journals, relevant journals and non-related journals by arranging all journals in descending order according to number of publications. The equation of Bradford’s law is:

(1) $$P=2\ln\left(E^{e}\cdot Y\right)$$

where P is the number of core journals; e is the Euler coefficient and equals 0.5772; *Y* is the number of papers in the most productive journal. 

In the SGD research, P=2ln(1.781×118)=10.7. And the 11 core journals were listed in Table 2, which had 746 publications with 40.46% of all 1,844 publications included. Marine Chemistry topped the list with 118 publications, accounting for 6.40% of total output. The following two were Journal of Hydrology (102, 5.53%), Estuarine Coastal and Shelf Science (80, 4.34%). Among the top 11 productive journals, Environmental Science & Technology had the highest 5-year impact factor (7.874), followed by Science of the Total Environment (5.727), Geochimica et Cosmochimica Acta (5.002), Water Resources Research (4.967), and Geophysical Research Letters (4.938). During 1998–2019, SGD research published on Marine Chemistry had the highest academic influence with 3915 total citations, followed by Journal of Hydrology (2830), Limnology and Oceanography (2648), Geochimica et Cosmochimica Acta (2448) and Water Resources Research (2396). The citation per publication (CPP) were all larger than 20 for the top 10 productive journals. In the perspective of *h*-index, Marine Chemistry had the highest *h*-index of 34, followed by Limnology and Oceanography (33) and Journal of Hydrology (30). All these core journals are located in JCR partition of Q1 and Q2, indicating the relatively high research quality in SGD research. 

### 3.4. Performance and Cooperation of Country/Territory

**Figure 4:**
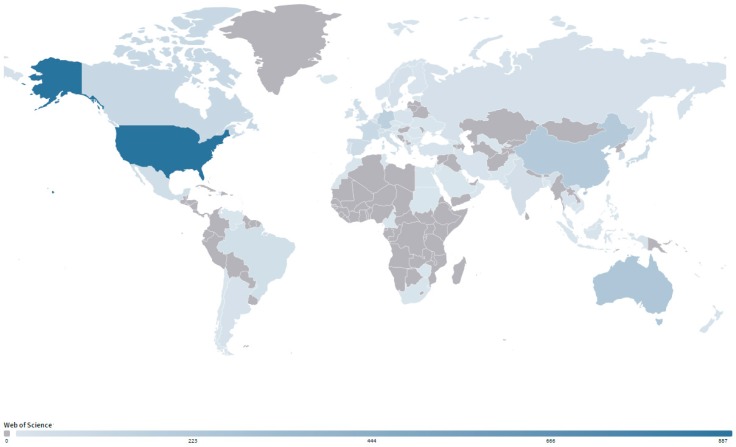
Geographic distribution of country/territory in SGD research.

Statistical analyses of country/territory performance and cooperation can not only help to find the most productive country/territory in the research field, but also get a clear understand of the output capacity of a country/territory and its difference with other countries/territories. Based on the author addresses, the geographic distribution of all 86 countries/territories that participated in SGD research was generated. As shown in Figure 4, most of them were located in coastal areas, in accordance with the marine-related research property of SGD. The USA ranked as the most productive country with an obvious advantage (887, 48.59%). Australia (238, 12.96%) ranked second, followed by China Mainland (206, 11.93%), Germany (161, 8.90%) and Canada (97, 5.32%). The top 5 productive countries/territories participated 73.59% of all research on SGD. Some implications were obvious from Table 3: first, the USA took a leading position in total output and outputs of first-author (716), corresponding-author (743) and international collaboration (324). Second, the USA was the major collaborative partner of the others except Switzerland, Taiwan and Ireland among the 20 countries/territories. Third, Sweden and Netherlands had high CPP in spite of a few publications, while India and Ireland had relatively low CPP. Moreover, China Mainland and Germany had relatively low CPP though with high research outputs, suggesting that their academic influence could be further strengthened by improving paper quality and visibility [35]. 

There were 606 international collaborative publications, accounting for 32.86% of totaled 1844 papers. The percentage of international collaboration on SGD research was apparently higher than other fields such as 14% in biosorption technology on water treatment [36], 16% in desalination research [37], and 23.8% in river water quality assessment and simulation [27]. For the top 20 productive countries/territories, at least half of publications were done with international collaborations except the USA, South Korea, India and Israel. Switzerland received largest contribution from collaboration (94%), followed by Netherlands (83%). These results indicated the character of active international connection in SGD research. 

**Figure 5:**
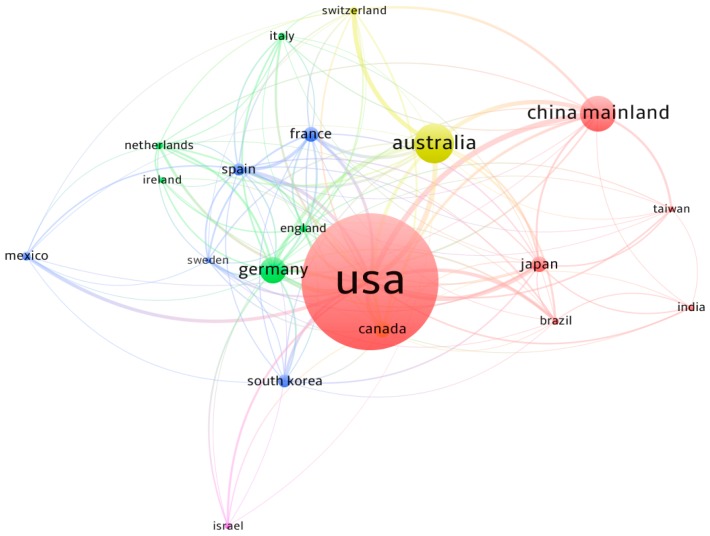
Visualization of international collaboration between the top 20 productive countries/territories.

Figure 5 showed the social network visualization of international collaborations between the 20 productive countries/territories. The size of circle for an item (e.g., one country/territory) presents the weight of the item. Links is an indicator in VOSviewer software, representing the number of connections of an item with other items. For example, the links of the USA is 19, indicating that it collaborated with the others 19 top productive countries/territories (see Table 3). In the collaborative network, the USA played a central position with the highest total collaboration intensity (LS = 368), suggesting its research capacity and potential in SGD research. The link strength between USA and China Mainland is maximum with 52 collaborative publications, followed by USA-Australia (51), USA-Germany (43), USA-Canada (36), and USA-Japan (29). In total, the USA collaborated with 55 countries/territories, followed by Germany with 45 partners, and Australia with 39 partners. It is worth noting that China and South Korea were located in comparatively low status in international collaborations when compared with the number of publications, with only 19 and 16 collaborative partners respectively. 

### 3.5. Research Performance of Institution

According to the literature analysis, there were 1403 institutions that participated in SGD research. The top 20 productive institutions, as shown in Table 4, completed 888 publications totally, accounting for 48% of total output. With eight of the top 20 institutions in the USA, the dominant position of the USA also extended to institutional level. The US Geology Survey was the most productive institution with 146 publications, followed by Florida State University (98), Woods Hole Oceanographic Institution (96), and University of South Carolina (80). Florida State University has the highest *h*-index of 41, followed by US Geology Survey (38). It is worth noting that there were 16 universities in the top 20 institutions. These results indicated that higher education academy played an important role in the development and innovation of SGD research.

Of the 1844 publications, 1238 were products of inter-institutional collaborations, with the percentage of 67%, which was higher than other fields such as 44% in solid waste research [23], and 62% in global climate change [33]. For the top 20 productive institutions, the percentages were also larger than or close to 50%. Collaboration outputs between CNRS and IRD ranked the first with 46 publications, followed by that between University of Queensland and Hohai University with 30 publications. These results suggested the character of active inter-institutional collaborations in SGD research.

### 3.6. Author Profile of Publications

Table 5 shows the top 10 productive authors in research on SGD. The total citations (TC), citations per publication (CPP), *h*-index, and the institution and country were also shown besides the number of total publications (TP). Of the 10 productive authors, 4 authors were from the USA, 3 from Australia, 1 from South Korea, Spain, China Mainland, and Japan, respectively. Santos IR, from Southern Cross University, was the most productive author with TP of 89, followed by Burnett WC (Florida State University; 76). Moore WS, from University of South Carolina, had the highest academic impacts with TC of 4673 and CPP of 65. Burnett WC and Moore WS had the highest *h*-index of 35. 

### 3.7. Hotspots and Research Trends

#### 3.7.1. The Performance of Author Keywords Analysis 

**Figure 6:**
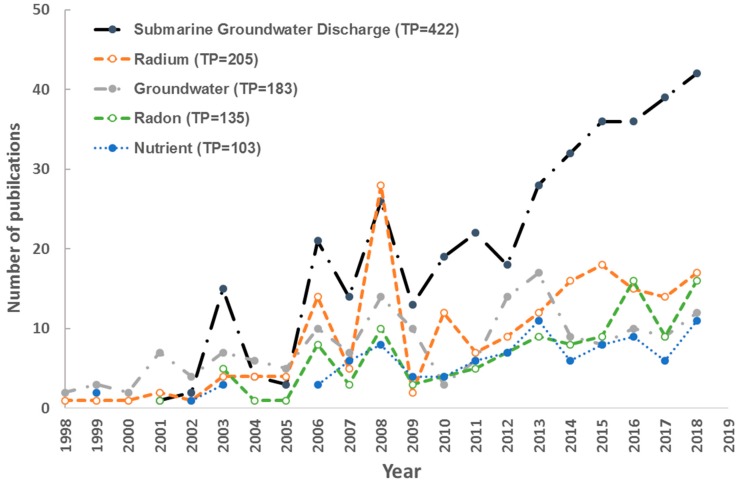
The growth dynamics of top 5 high-frequency keywords.

Author keywords can provide important information about the core content and research trends [26,31]. In this study, there were 3267 unique keywords that appeared 7029 times in the 1285 publications with non-empty author keywords field. Except the search terms “Submarine groundwater discharge” and “Groundwater”, “Radium” was the most frequently used keyword (205 times, 2.84% of total keywords occurrence), followed by “Radon” (135, 1.87%), “Nutrient” (103, 1.43%). Figure 6 illustrated the growth dynamics of the top five high-frequency keywords. “Nutrient” occurred continuously since 2006 with an obvious growth over the last decade, owing to the increasing emphasis on the marine environment and strengthening of environmental regulations (e.g., United Nations Convention on the Law of the Sea, Global Programme of Action for Protection of Marine Environment from Land-based Activities). “Radium” and “Radon” have become increasing important as two important geochemical tracers in SGD quantification. Overall, the study of radium developed faster than that of radon in the early stage, but no obvious difference of publication outputs was observed in recent years.

#### 3.7.2. Co-Words Cluster Visualization

Cluster analysis can not only help to gather related keywords, but also reflect the close relationship between keywords. In this study, Vosviewer software [38] was applied to identify the mainstream research with cluster analysis of author keywords and Keywords plus. 

**Figure 7:**
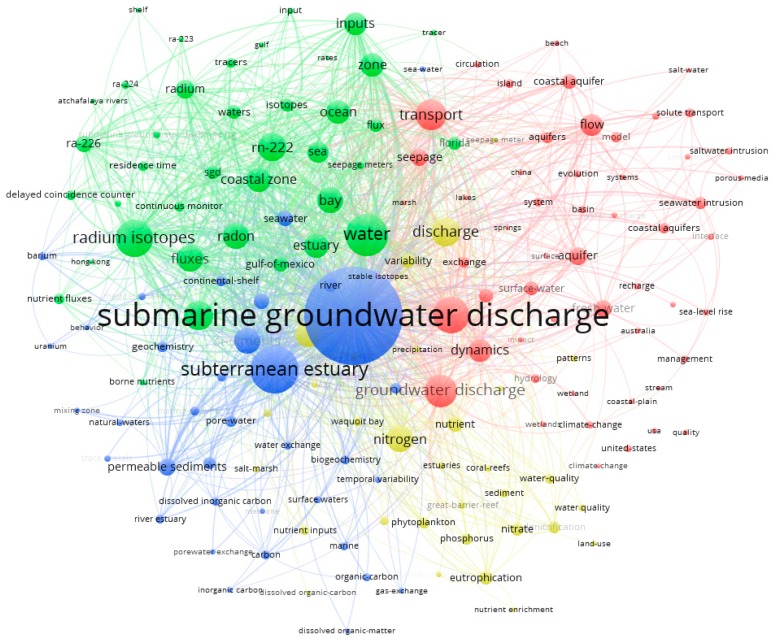
The results of co-words cluster analysis.

According to the result of cluster analysis as shown in Figure 7, all keywords were aggregated into four clusters. Cluster 1, the largest cluster with red items, involved the dynamics process and estimation of SGD with hydrogeological methods, including hydrogeologic terminology such as “aquifer”, “transport”, “porous media”, “solute transport”, “model” and “seawater intrusion”. Cluster 2, with green items, focused on tracer techniques to estimate SGD with keywords including “isotopes”, “radium”, “radon”, “delayed coincidence counter”, “residence time”, and “continuous monitor”. There are various isotopes of radium and radon including “Rn-222”, “Ra-226”, “Ra-224”, “Ra-223” etc. Cluster 3, with blue items, referred to the geochemical process in subterranean estuary (i.e., the mixing and action zone of groundwater and seawater), with keywords of “subterranean estuary”, “geochemistry”, “permeable sediments” occurred. Cluster 4, with yellow items, focused on dissolved materials inputs to coastal waters via SGD, including the main keywords of “nitrogen”, “nutrient”, “nitrate”, “eutrophication” and “water quality”. 

#### 3.7.3. The Citation Analysis of Hot Publications

HistCite was used to identify hot publications with citations analysis. Global citation score (GCS) and local citation score (LCS) are two important parameters in HistCite. GCS refers to the total citations (TC) in Web of Science database. LCS is the citation times from papers in the import database of HistCite. Since those papers imported into HistCite were SGD-related publications, papers with high LCS are key literatures in SGD research. Take a paper with high GCS but low LCS for example, high GCS indicates high attentions of scholars around the world, but low LCS suggests that this paper is mainly focused by scholars in different fields with SGD research. 

The paper with the highest academic influence in SGD research field was “The subterranean estuary: a reaction zone of ground water and sea water”, written by Moore WC (1999) [39] with LCS of 405 and TC of 550. Moore’s other paper “The Effect of Submarine Groundwater Discharge on the Ocean”, published in 2010 [3], belonged to the category of highly cited papers in ESI database. 

**Figure 8:**
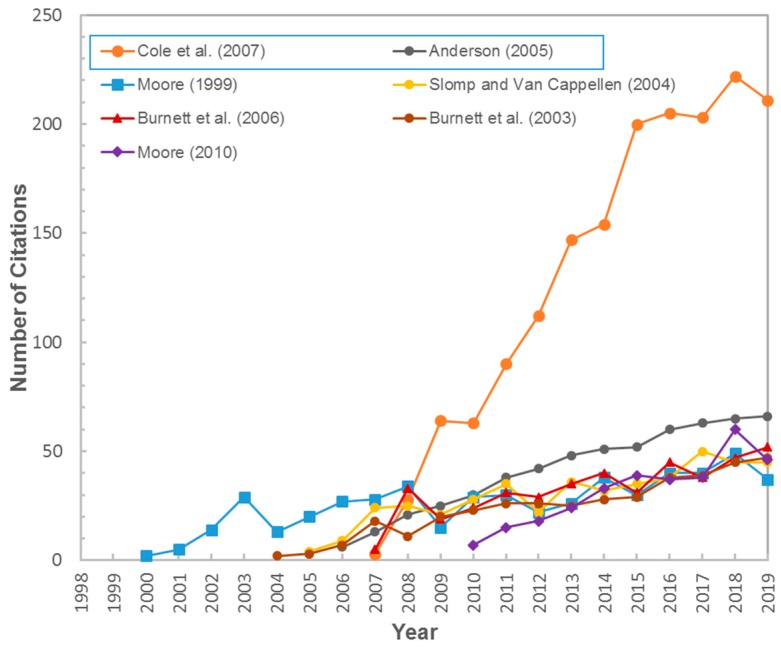
The growth dynamics of citations for the top 5 high LCS and two high TC publications.

Through comparing between the top five publications with high LCS and those with high TC, there were two papers, Cole et al. (2007) [40] and Anderson (2005) [41], had high TC but relatively low LCS. In other words, their citations were mainly from researches in other fields rather than the SGD field. Specifically, “Plumbing the global carbon cycle: Integrating inland waters into the terrestrial carbon budget” published in Ecosystems by Cole et al. was mainly cited to study carbon cycle, greenhouse gases and global warming. Further, “Heat as a ground water tracer”, published in Ground Water by Anderson, was mainly cited by researches of groundwater heat transport, heat storage of aquifer system, groundwater-surface water exchange, and hot springs. Previous studies showed that SGD is an important pathway for carbon flux into the marine environment, while there are only a few related studies at present [3,42]. Similarly, the usage of head as groundwater tracer is currently far from being fully utilized [43,44,45]. As shown in Figure 8, the citations of the two papers increased significantly, with TC increased to 211 and 66 in 2019 respectively. These results indicated the good development space and high possibility of making great breakthroughs in these two topics by further efforts. 

## 4. Conclusions 

This study presented a systematic bibliometric analysis of literatures on submarine groundwater discharge (SGD) based on Web of Science database during 1998–2019. Results showed the scientific output in SGD research increased rapidly in the last several decades, and the cumulative number of publications fits Price’s curve. These 1844 publications on SGD research covered 55 subject categories. Environmental Sciences, Water Resources, Geosciences Multidisciplinary, Oceanography and Marine & Freshwater Biology were the top five main subject categories. Multi-subject publications accounted for 61% of total outputs, indicating the character of active multi-discipline cross in SGD research. The research on SGD published on 310 academic journals, with the number of journals increasing linearly. There were 11 core journals published in the field of SGD, including Marine Chemistry, Journal of Hydrology, Estuarine Coastal and Shelf Science, Geochimica et Cosmochimica Acta, Water Resources Research, Limnology and Oceanography, Science of the Total Environment, Continental Shelf Research, Environmental Science & Technology, Geophysical Research Letters, Estuaries and Coasts. USA had distinct advantages in publication outputs including the first-author, corresponding-author, international collaborations and the total. Meanwhile, the USA played a core role in the collaborative network with another 55 countries. By contrast, the scientific quality and international collaboration of China and South Korea need to be further improved. The US Geology Survey, Florida State University, the Woods Hole Oceanographic Institution, the University of South Carolina, and Southern Cross University were the top five productive institutions. It was indicated that higher education academies played a significantly important role in the SGD research. The frequent activities were identified in international and inter-institutional collaborations. Santos IR, Burnett WC, and Moore WS were the top three productive and influential scholars in SGD research. 

From the analysis of author keywords, “Radium”, “Radon” and “Nutrient” were the most high-frequency keywords except the search terms “Submarine groundwater discharge” and “Groundwater”. Research hotspots of SGD mainly include the following: dynamics process and estimation of SGD with hydrogeological methods, tracer techniques, geochemical process in subterranean estuary, and dissolved material inputs to coastal waters via SGD. The comparison between local citation score and total citation times in Histcite software showed that far from sufficient research had been made in carbon flux transported by SGD and the usage of head as groundwater tracer. These two topics could be made of great breakthrough with strong academic influences in the future by further efforts. 

Based on the bibliometric analyses, one can see that radium and radon are two important geochemical tracers used in SGD quantification, and have gradually become the dominant research approach nowadays. However, how to reduce the common uncertainty in radium and radon mass balance models associated with selecting groundwater end-member is an important but difficult task. Secondly, the impacts of SGD on environment and water resource has attracted growing emphasis, since the dissolved materials from SGD could be comparable to those inputs from rivers. Nevertheless, it is difficult to separate inland freshwater from total SGD based on the present technology. Besides that, many studies did not couple the exchange in water quantity (mass of water) and water quality (mass of various chemicals) between seawater and groundwater. Hence, a numerical model coupling the exchange in water quantity and quality would be continuously encouraged to address in future research. 

## Figures and Tables

**Table 1:** Characteristics of the top 10 hot subject categories.

Subject Category	TP	TC	Multi-Subject		MS (P)
			TP	%	
Environmental Sciences	563	11,667	439	78	Water Resources (173)
Water Resources	484	11,878	393	81	Geosciences, Multidisciplinary (381)
Geosciences, Multidisciplinary	474	11,368	386	81	Water Resources (258)
Oceanography	459	12,879	353	77	Marine & Freshwater Biology (138)
Marine & Freshwater Biology	292	6417	270	92	Oceanography (138)
Geochemistry & Geophysics	186	5914	12	6	Marine & Freshwater Biology (4); Oceanography (4)
Limnology	174	5438	171	98	Oceanography (89)
Chemistry, Multidisciplinary	127	4338	118	93	Oceanography (118)
Engineering, Civil	120	3005	120	100	Water Resources (102)
Engineering, Environmental	74	1722	74	100	Environmental Sciences (67)

TP: the number of publications; TC: total citations; %: percentage of multi-subject publications; MS (P): major intercrossed subject (the number of multi-subject publications between two subject categories).

**Table 2:** Characteristics of the 11 core journals in the research on SGD.

Journal	TP	%	IF5	JCR Partition	TC	CPP	*h*-Index
Marine Chemistry	118	6.40	3.779	Q2	3915	33.2	34
Journal of Hydrology	102	5.53	4.938	Q1	2830	27.7	30
Estuarine Coastal and Shelf Science	80	4.34	2.975	Q1	1686	21.1	23
Geochimica et Cosmochimica Acta	73	3.96	5.002	Q1	2448	33.5	27
Water Resources Research	73	3.96	4.967	Q1	2396	32.8	28
Limnology and Oceanography	68	3.69	4.402	Q1	2648	38.9	33
Science of the Total Environment	51	2.77	5.727	Q1	1178	23.1	16
Continental Shelf Research	48	2.60	2.494	Q2	1020	21.3	18
Environmental Science & Technology	46	2.50	7.874	Q1	1335	29	21
Geophysical Research Letters	44	2.39	4.909	Q1	1444	32.8	20
Estuaries and Coasts	43	2.33	2.883	Q1	517	12	15

TP: the number of publications; IF5: 5-year impact factor; TC: total citations; CPP: citations per publication; *h*-index: defined by the *h* of TP papers having at least *h* citations each and the other (TP-*h*) papers having ≤*h* citations each [28].

**Table 3:** Characteristics of the top 20 productive countries/territories.

Country/Territory	TP	FP (%)	RP (%)	ICP (%)	MC (P)	CPP	*h*-Index	Links
USA	887	716 (81)	743 (84)	324 (37)	China Mainland (52)	33.23	77	19
Australia	238	144 (61)	146 (61)	141 (59)	USA (51)	30.1	42	17
China Mainland	206	182 (88)	181 (88)	103 (50)	USA (52)	15.2	28	13
Germany	161	97 (60)	106 (66)	114 (71)	USA (43)	17.48	27	19
Canada	97	51 (53)	54 (56)	70 (72)	USA (36)	35.8	24	15
France	90	47 (52)	53 (59)	62 (69)	USA (22)	17.4	21	13
Japan	90	62 (69)	61 (68)	46 (51)	USA (29)	27.51	24	15
South Korea	74	65 (88)	64 (86)	17 (23)	USA (12)	19.97	21	11
Spain	72	46 (64)	43 (60)	57 (79)	USA (21)	39.33	21	18
Mexico	51	32 (63)	32 (63)	29 (57)	USA (22)	18.94	18	9
England	45	19 (42)	17 (38)	33 (73)	USA (13)	25.49	17	16
Italy	44	23 (52)	23 (52)	32 (73)	USA (9)	23.64	15	11
Netherlands	42	17 (40)	17 (40)	35 (83)	USA (13)	75.95	17	12
Brazil	42	19 (45)	19 (45)	32 (76)	USA (23)	26.76	16	13
India	40	35 (88)	34 (85)	14 (35)	USA (5)	4.6	7	14
Switzerland	35	12 (34)	13 (37)	33 (94)	Australia (18)	40.77	19	12
Israel	32	25 (78)	23 (72)	15 (47)	USA (10)	18.66	13	6
Taiwan	28	16 (57)	16 (57)	22 (79)	China Mainland (10)	18.39	11	9
Ireland	28	22 (79)	20 (71)	14 (50)	Netherlands (4)	9.36	9	14
Sweden	28	16 (57)	16 (57)	17 (61)	USA (6)	81.54	17	13

TP: the number of publications; FR (%): the number and percentage of first-author country/territory publications, RP (%): the number and percentage of corresponding-author country/territory publications; ICP (%): the number and percentage of international collaborative publications, MC (P): major collaboration partner (the number of collaborative publications between two countries/territories); CPP: citations per publication.

**Table 4:** Characteristics of the top 20 productive institutions.

Institution	Country	TP	ICP (%)	MC (P)	*h*-Index
US Geol Survey	USA	146	111 (76)	Woods Hole Oceanog Inst (13)	38
Florida State Univ	USA	98	77 (79)	RIHN (14)	41
Woods Hole Oceanog Inst	USA	96	75 (78)	Univ S Carolina (15)	36
Univ S Carolina	USA	80	66 (83)	Woods Hole Oceanog Inst (12)	37
So Cross Univ	Australia	74	34 (46)	Carl Von Ossietzky Univ Oldenburg (7)	26
CNRS	France	65	64 (98)	IRD (46)	17
Seoul Natl Univ	Korea	53	26 (49)	Pukyong Natl Univ (5)	21
IRD	France	50	48 (96)	CNRS (46)	15
N Carolina State Univ	USA	45	36 (80)	E Carolina Univ (13)	17
Univ Queensland	Australia	42	41 (98)	Hohai Univ (30)	20
Univ Florida	USA	40	35 (88)	Louisiana State Univ (13)	18
China Univ Geosci	China	40	30 (75)	South Univ Sci & Technol China (11)	12
Hohai Univ	China	37	37 (100)	Univ Queensland (30)	16
Xiamen Univ	China	37	26 (70)	Univ S Carolina (8)	12
Univ Autonoma Barcelona	Spain	37	34 (92)	CNRS (12)	16
Stanford Univ	USA	36	27 (75)	US Geol Survey (6)	20
Univ Delaware	USA	33	17 (52)	US Geol Survey (6)	17
E China Normal Univ	China	33	25 (76)	Ocean Univ China (7)	11
Flinders Univ S Australia	Australia	33	27 (82)	CNRS (6)	13
Carl Von Ossietzky Univ Oldenburg	Germany	32	22 (69)	So Cross Univ (7)	16
UFZ	Germany	32	26 (81)	Univ Kiel (6)	12

TP: the number of publications; ICP (%): the number and percentage of publications with inter-institutional collaboration, MC (P): major collaborative institution (the number of collaborative publications between two institutions).

**Table 5:** The top 10 productive authors and their academic impacts.

Author	Institute	Country	TP	TC	CPP	*h*-Index
Santos IR	So Cross Univ	Australia	89	2782	31	31
Burnett WC	Florida State Univ	USA	76	4415	58	35
Moore WS	Univ S Carolina	USA	72	4673	65	35
Charette MA	Woods Hole Oceanog Inst	USA	53	3051	58	31
Kim G	Seoul Natl Univ	South Korea	45	1523	34	21
Maher DT	So Cross Univ	Australia	41	1034	25	19
Garcia-Orellana J	Univ Autonoma Barcelona	Spain	35	797	23	18
Li L	Univ Queensland	Australia	34	1510	44	20
Taniguchi M	Res Inst Humanity & Nat	Japan	34	2266	67	21
Li HL	China Univ Geosci	China Mainland	31	400	13	13
Swarzenski PW	US Geol Survey	USA	31	1178	38	21

TP: the number of publications; TC: total citations; CPP: citations per publication.
